# Alterations in Biomarkers Associated with Cardiovascular Health and Obesity with Short-Term Lifestyle Changes in Overweight Women: The Role of Exercise and Diet

**DOI:** 10.3390/medicina60122019

**Published:** 2024-12-07

**Authors:** Nezihe Şengün, Ragıp Pala, Vedat Çınar, Taner Akbulut, Alin Larion, Johnny Padulo, Luca Russo, Gian Mario Migliaccio

**Affiliations:** 1 Department of Nutrition and Dietetics, Faculty of Health Sciences, Istanbul Kültür University, Istanbul 34158, Turkey; n.sengun@iku.edu.tr; 2 Department of Coaching Education, Faculty of Sports Science, Fırat University, Elazig 23119, Turkey; rpala@firat.edu.tr (R.P.);; 3 Department of Physical Education and Sport, Faculty of Sports Science, Fırat University, Elazig 23119, Turkey; cinarvedat@hotmail.com; 4 Faculty of Physical Education, Ovidius University of Constanta, 900029 Constanta, Romania; larion.alin@univ-ovidius.ro; 5 Department of Biomedical Sciences for Health, Università degli Studi di Milano, 20133 Milan, Italy; johnny.padulo@unimi.it; 6 Department of Theoretical and Applied Sciences, eCampus University, 22060 Novedrate, Italy; 7 Department of Human Sciences and Promotion of the Quality of Life, San Raffaele Rome Open University, 00166 Rome, Italy; 8 Athlete Physiology, Psychology and Nutrition Unit, Maxima Performa, 20126 Milan, Italy

**Keywords:** weight loss, exercise, nutrition, health, GDF15

## Abstract

*Background and Objectives*: In this study, the effects of an eight-week exercise and nutrition program on blood lipids, glucose, insulin, insulin resistance (HOMA-IR), leptin, ghrelin, irisin, malondialdehyde (MDA), and Growth Differentiation Factor 15 (GDF15) in overweight women were investigated. *Materials and Methods*: A total of 48 women volunteers participated in this study. The participants were randomly divided into four groups: control (C), exercise (E), nutrition (N), exercise + nutrition (E + N). While no intervention was applied to group C, the other groups participated in the predetermined programs for 8 weeks. At the beginning and end of this study, body composition was measured and blood samples were taken. *Results*: It was determined that the body composition components, lipid profile indicators, insulin, glucose, insulin resistance, leptin, ghrelin, irisin, and MDA parameters examined in this study showed positive changes in the intervention groups. Group E had a greater effect on body muscle percentage, MDA, and irisin levels, while group N had a greater effect on blood lipids and ghrelin levels. *Conclusions*: As a result, it is thought that lifestyle changes are important to improve cardiovascular health and combat obesity, and that maintaining a healthy diet together with exercise may be more effective.

## 1. Introduction

Today, obesity is one of the most important health problems in the developing world and is the most important cause of the increase in morbidity/mortality rates [1]. Excessive or insufficient energy intake and the imbalance occurring as a result of insufficient energy expenditure are among the factors causing obesity [2,3]. It is well known that women are more prone to obesity due to their lifestyle and physiology [4]. Regular exercise is one of the factors determining weight loss and fat loss and is also associated with significant health benefits [5]. Aerobic exercise programs are known as one of the most important components in obesity treatment [6,7,8,9]. The World Health Organization recommends at least 150 min of exercise per week for adults aged 18–65 in its published report [10]. The American College of Sports Medicine recommends that all healthy adults partake in at least 30 min of moderate-intensity aerobic exercise 5 days a week to improve and maintain their health [11]. It is recommended that healthy body weight loss can be achieved with exercise habits and 30 min of aerobic exercise at 60–75% intensity 3 days a week [10,11]. BMI does not provide information about fat distribution, which is of high importance for cardiovascular risk [12]. Overweight and obesity are associated with an increased risk of cardiovascular disease [13].

Obesity is a major contributor to dysfunction of the liver, heart, lung, endocrine, and reproductive systems and is a component of metabolic syndrome. Although the development of obesity-related disorders is associated with lipid abnormalities, most previous studies on this issue included routinely determined parameters such as triacylglycerol, total cholesterol, and serum concentrations of low-density and high-density lipoprotein cholesterol [14]. The increase in fat mass in adipose tissue plays a role in the development of insulin resistance along with obesity [15].

Insulin resistance is defined as an abnormal glucose response to exogenous and endogenous insulin. In large population studies, insulin resistance is measured with simple formulas using fasting insulin and glucose values. HOMA-IR (homeostasis model assessment of insulin resistance) is one of the widely used methods [16]. It was reported that healthy nutrition and performing exercise are effective strategies to improve metabolic abnormalities in slightly overweight and obese individuals [17]. Circulating peptides such as leptin and ghrelin, which have an effect on appetite, have been associated with adiposity [1,18]. İrisin is traditionally considered a myokine involved in the browning of white adipose tissue, energy expenditure, and glucose tolerance. Its link to fat accumulation and metabolic dysfunction is debated. It is debated whether it has a link with fat accumulation and metabolic dysfunction [19].

Growth Differentiation Factor 15 (GDF15) is a stress response cytokine secreted into circulation [20,21]. A decrease in food intake occurs due to an increase in GDF15 secretion, or conversely, an increase in food intake occurs due to a decrease in GDF15 levels; therefore, it is thought to have a metabolic effect [22]. At the systemic level, excessive reactive oxygen species (ROS) production associated with nutritional imbalance is among the main determinants of overall deterioration in health status due to obesity [23,24]. Malondialdehyde (MDA) is used as an indicator of oxidative stress. The concentration of MDA, a serum lipid peroxidation product, is measured to determine the level of lipid peroxidation [25].

Studies tend to focus particularly on obese individuals, but overweight individuals are ignored in this regard. Therefore, the number of studies on overweight individuals is limited and more research is needed. In the present study, it was hypothesized that in order to prevent obesity and predict it with biomarkers, the effects of lifestyle changes on cardiovascular health and obesity-related biomarkers in overweight women could be determined, thus enabling both diagnosis and preventive health actions. Therefore, the aim of this study was to determine the effects of an eight-week personalized nutrition and exercise intervention applied to overweight women on TC, HDL, LDL, TG, glucose, insulin, HOMA-IR, ghrelin, leptin, irisin, GDF15, MDA, and body composition.

## 2. Materials and Methods

### 2.1. Study Group

**Figure 1:**
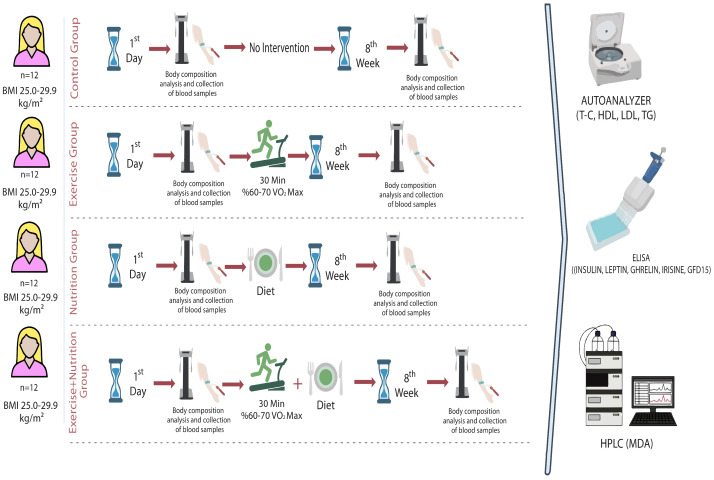
Research design.

This study consisted of 48 sedentary women with a median age of 24 (18–28) and a BMI between 25.0 and 29.9 kg/m^2^ who provided an informed consent form. The study was conducted in accordance with the pre-test/post-test model with a control group. In this context, an exercise and nutrition program was applied to the study groups for 8 weeks (Figure 1). Before starting the study, ethics committee approval was obtained from the Fırat University Non-Interventional Research Ethics Committee with session date 16 September 2021 and session number 2021/09-53. Additionally, the study was conducted in accordance with the Declaration of Helsinki. The sample size in the study was determined to be 48 people (n = 12; 4 groups) in total with an effect size of 0.55, alpha error of 0.05, and G*Power package program at 85% power (version 3.1.9.7) (Table 1) [26].

### 2.2. Body Composition Analyses

Bioelectrical Impedance Analysis (BIA) was measured using the Tanita BC601 Fast Body Analyzer (Amsterdam, The Netherlands). Waist and hip circumferences were measured with a tape measure. The ratio of waist and hip circumferences to each other was calculated using the obtained circumference measurements (waist/hip). Possible changes (energy and nutrients) were made to the nutrition program according to changes in body weight, and a personalized re-preparation was made.

### 2.3. Exercise

In the meta-analysis conducted by Wirth et al. [27], it was reported that creating a 500 kcal energy deficit through exercise would be sufficient. Additionally, since healthy body weight loss can be achieved through exercise habits, 30 min of aerobic exercise at 60–75% intensity 3 days a week is recommended. In this study, the participants in groups 2 and 4 were made to run at 60–70% intensity using the Karvonen formula 5 days a week for 8 weeks [28,29]. The first week was considered the acclimatization period, and exercises were performed at 50% intensity. A total of 10 min of warm-up exercises, 30 min of running (Voit Epic II Plus treadmill) at 60–70% intensity using the Karvonen formula, and 10 min of cooling down and stretching exercises were performed. A heart rate sensor kept track of the hearth rates before, during, and after the treadmill.

### 2.4. Personalized Nutrition Program

Healthy nutrition is the intake of sufficient and balanced energy and nutrients for the body and is essential for the protection and sustainability of health [30,31]. In our study, a nutrition program was prepared according to the energy and nutrient needs of group 3 and group 4 participants for 8 weeks. Since energy and nutrient needs vary from person to person, the eating habits, physical activity level, gender, age, BMI, and disease factor of individuals were taken into account when preparing a healthy nutrition program [32]. Physical activity level, total energy expenditure, and dietary energy were calculated [30,31]. The basal energy requirements of the participants were calculated using the Harris–Benedict formula [33]. The nutrition program was designed according to obesity medical nutrition therapy, so that 50–55% of the energy would come from carbohydrates, 12–15% from protein, and 25–30% from fat [30,31]. The nutrition programs of the women who would implement them were organized by taking care not to reduce the energy needs of the participants below 1200 kcal so that their health would not be negatively affected, i.e., so that they would not have any vitamin and mineral deficiencies [34]. According to the data of the Türkiye Nutrition Guide (TÜBER), the daily water requirement for the people in our study group was determined as 35 mL × body weight [35]. Twenty-four-hour food consumption records of the participants in group 3 and group 4 were taken. In order for the participants to measure their portion sizes more accurately, the book *Food and Nutrient Photograph Catalogue Measurements and Amounts* developed by Rakıcıoğlu et al. (2014) was used [36]. In the evaluation of daily energy and nutrients taken, the “Computer Assisted Nutrition Program, Nutrition Information System-BEBIS (9.0)” program developed for Türkiye was used.

According to the results obtained from the food consumption records at the beginning of this study, it was seen that the participants consumed more calories than their daily energy needs. In this context, the creation of a nutrition program according to the participants’ individual energy needs revealed a non-fixed calorie restriction method. Since the nutrition program was personalized, no fixed calorie restriction was applied. In addition, the participants’ food preferences were taken into consideration in order to increase their compliance with the nutrition program. Weekly body weight checks were made. During the checks, the nutrition programs were updated according to their energy needs.

### 2.5. Taking Blood Samples

Blood samples were taken twice, at the beginning (day 1) and at the end (day 60), as 5 mL between 08:00 and 08:30 in the morning on an empty stomach after a 12 h overnight fast, avoiding any kind of caffeine [37]. The blood samples taken were placed in gel biochemistry tubes (Standardplus&Medical Co., Ltd., Düsseldorf, Germany). After being centrifuged in a refrigerated centrifuge (Universal 320R, Hettich, Kirchlengern, Germany) at 5000 rpm and 4 °C for 10 min, the serum samples obtained were placed in Eppendorf tubes and stored in a deep freezer (Hettich, Kirchlengern, Germany) at −80 °C until they were analyzed.

### 2.6. Homeostatic Model Assessment of Insulin Resistance (HOMA-IR)

The HOMA-IR value was calculated as (1), and the patients with a HOMA score ≥ 2.7 were considered insulin resistance (IR)-positive [16].

HOMA-IR = Fasting Glucose(mg/dL) × Fasting Insulin(uIU/mL)/405(1)

### 2.7. Serum Analyses

Serum biochemical parameters, namely total cholesterol, HDL, LDL and triglyceride, and glucose levels, were analyzed using an autoanalyzer (Samsung Labgeo PT10); insulin (catalog no: 201-12-0011), leptin (catalog no: 201-12-1560), ghrelin (catalog no: 201-12-5583), irisin (catalog no: 201-12-5328), and GDF15 (catalog no: 201-12-0038) levels were analyzed using commercial kits (EasyStep) (ELISA, Köln, Germany) specifically for humans (BIO-TEK ELX800); and MDA was analyzed using the HPLC technique known as high-performance liquid chromatography or high-pressure liquid chromatography [38].

### 2.8. Statistical Analysis

The SPSS statistical package program (IBM SPSS version 22.0) was used to evaluate the data [39]. In this study, compliance with the normality assumption, which is one of the prerequisites of parametric tests for the data, was checked with the “Shapiro–Wilk” test, and the homogeneity of variances was checked with the “Levene” test. Accordingly, parametric or non-parametric tests were used. For parametric data, a one-way analysis of variance (ANOVA) was used to determine the differences between the groups, and the “Duncan” test was used as a post hoc analysis in pairwise comparisons of the groups. While the “Kruskal–Wallis” test was used for variance analysis of the groups for which the normality assumption could not be provided, pairwise comparisons of the groups were evaluated with the “Mann–Whitney U” test. To reveal the changes in the data over time (before–after) for each group, the “dependent samples t test” was used for data showing normal distribution, while the “Wilcoxon test” was used for data not showing normal distribution. Correlation analysis was performed to provide information about the relationship between variables and the direction and intensity of this relationship. The level of statistical significance was accepted as *p* < 0.05.

## 3. Results

The change in body weight over the 8-week period was found to be significant (*p* ≤ 0.0001). Compared to group C, the BMI changes in other groups were significant (*p* ≤ 0.0001). A significant decrease in body fat percentage was detected over the 8-week period (*p* ≤ 0.0001). It was determined that the greatest decrease in body fat percentage was in group E + N. When body muscle percentage changes were examined, the change between the groups was significant (*p* = 0.004). When the degree of visceral fat was examined, the difference between the before and after of all the groups except group C was found to be significant (*p* < 0.05). It was determined that there was significance between the pre- and post-waist–hip ratio values of the group E + N (*p* = 0.004, Table 2).

In this study, the change in the TC and HDL levels was found to be significant (*p* ≤ 0.0001). The highest increase in HDL cholesterol levels occurred in group E + N (~16%). No significant difference was found between LDL cholesterol changes in all groups (*p* = 0.235). It was determined that there was no significant difference between the TG changes in all groups (*p* = 0.322), and there was a decrease in the TG levels in all groups except the control group (*p* > 0.05). It was found that there was a significant difference between the groups in the pre- and post-changes in glucose, insulin, and HOMA-IR levels. Additionally, a significant decrease was detected in glucose, insulin, and HOMA-IR levels in group N (*p* < 0.05, Table 3).

When insulin changes in this study were examined, a significant difference was found between the insulin changes in all groups (*p* ≤ 0.0001). There was a significant difference between the before leptin levels according to the 8-week period (*p* = 0.045). The difference between the after leptin levels of the groups over the 8-week period was significant (*p* = 0.027). There was a significant difference in the ghrelin levels in group N before and after the 8-week period (*p* = 0.003). When irisin changes were examined, it was determined that the change between the groups was significant (*p* ≤ 0.0001). There was a significant difference between the before GDF15 levels according to the 8-week period (*p* ≤ 0.0001). The difference between after GDF levels of the groups according to the 8-week period was significant (*p* = 0.018). When MDA changes were examined, no significant difference was found between the groups, but there was significance between the before and after MDA levels in group E (*p* = 0.018, Table 4).

**Figure 2:**
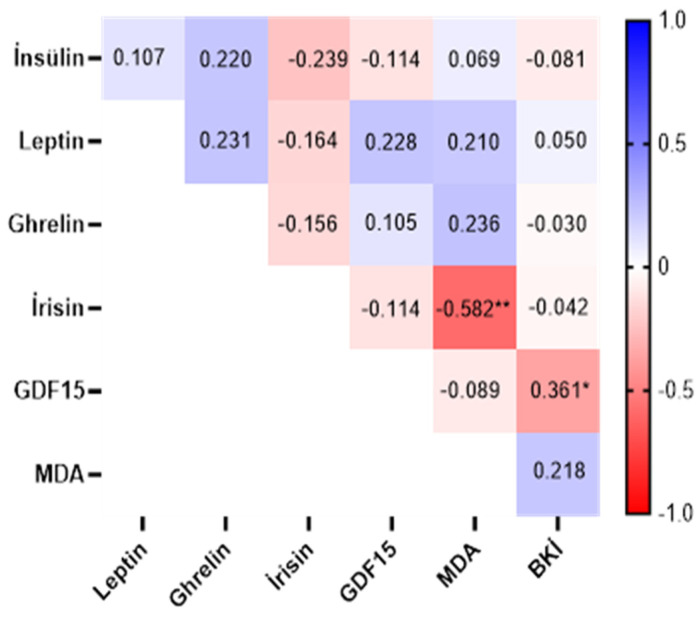
Correlation between some serum parameters (Insulin (μIU/mL), Leptin (ng/mL), ghrelin (pg/mL), irisin (ng/mL), GDF15: Growth Differentiation Factor 15 (ng/L), MDA: malondialdehyde (nmol/mL).

The correlation between some serum parameters is given in Figure 2. The correlation between irisin and MDA was found to be negative and moderately strong (r = −0.582, *p* < 0.01). The correlation between GDF15 and BMI was found to be negative and weakly strong (r = −0.361, *p* < 0.05, Figure 2).

## 4. Discussion

In the present study, it was hypothesized that in order to prevent obesity and predict it with biomarkers, the effects of lifestyle changes on cardiovascular health and obesity-related biomarkers in overweight women could be determined, thus enabling both diagnosis and preventive health actions. Therefore, the aim of this study was to determine the effects of an eight-week personalized nutrition and exercise intervention applied to overweight women on TC, HDL, LDL, TG, glucose, insulin, HOMA-IR, ghrelin, leptin, irisin, GDF15, MDA, and body composition.

It was reported that performing exercise, in addition to calorie restriction through diet, was more effective in weight loss [40], and a monthly body weight loss of 4 to 6 kg could be achieved with only nutritional therapy and 2 to 3 kg with only exercise therapy [27]. In this study, the greatest body weight loss occurred in group E + N, while the least body weight loss occurred in group N. Accordingly, it was shown that effective body weight loss is possible with the combined application of exercise and healthy nutrition. The amount of daily calories people consume is important in the energy deficit that is attempted to be achieved through the diet and is directly proportional to their weight loss. In this study, calorie restriction was calculated according to the daily energy needs of individuals in order not to cause vitamin–mineral deficiencies and negatively affect health. Therefore, it can be seen that there are differences in terms of body weight losses between studies conducted only on calorie restriction and this study. As stated by the American Association of Clinical Endocrinologists and the American College of Endocrinology “Clinical Practice Guidelines,” it is recommended that an initial body weight loss goal of 5% to 10% of the initial weight within 6 months is set, combined with the provision of a comprehensive lifestyle intervention [41]. When we evaluate this study from this perspective, it can be understood that the E + N group lost approximately 7% of their mean initial weight in an 8-week period. It can be said that performing exercise and having a healthy nutrition together can have a faster and greater effect on reaching the target weight. In their study, conducted in 2019, Seo et al. reported that a 16-week nutrition and exercise intervention had no effect on the additional BMI reduction in slightly obese adolescents [42], while in the study conducted in 2017, Correa-Rodríguez et al. reported that the diet factor was more effective than exercise on BMI and provided a more significant decrease [43]. In this study, BMI decreased in all groups except group C. The largest BMI difference between the groups occurred in group E + N. Although the BMI method has benefits, it is anticipated that not all individuals at risk for obesity-related medical conditions are identified [44]. The classification of obesity depending on BMI remains limited [45]. In this context, it was observed that the individuals who were slightly obese/overweight according to the most commonly used BMI classification in the classification of obesity at the beginning of this study were still in the slightly obese/overweight category at the end of the 8 weeks. BMI classification is thought to have wide ranges. The body fat index was found to be more sensitive than BMI in defining and classifying obesity [46]. The largest difference in body fat percentage between the groups in this study occurred in group E + N. It is stated that the combination of exercise and healthy nutrition practices may be more effective in reducing body fat percentage. Exercise provides an increase in muscle mass by regulating metabolism rather than losing body weight [47]. Consuming adequate dietary protein is critical for maintaining optimal health, growth, development, and function throughout life. The minimum amount of dietary protein required to prevent muscle mass loss must be met [48]. When muscle mass was examined as a percentage, a significant increase was observed in group E and group E + N. In this context, it can be said that exercise has an effect of increasing muscle mass. According to the classification of the waist–hip ratio, being greater than 0.90 in men and 0.85 in women is one of the diagnostic criteria of obesity by the WHO [49]. In this study, it was observed that the decrease in the waist–hip ratio occurred the most in group E + N. Accordingly, it is thought that the decrease in the degree of visceral fat in the E + N group is supported in this study. While the women in this group were considered to be in the risk group when looking at their waist–hip ratio at the beginning of this study, they were classified as being out of the risk group as a result of the combined application of exercise and nutrition.

It was reported that exercise intervention has a reducing effect on blood lipid levels [50]. According to the results of a meta-analysis consisting of 80 studies including 4804 adult participants investigating the effects of exercise, diet, and exercise + diet interventions on blood lipids, it was reported that exercise application was more effective in reducing TC and TG compared to diet application, diet application was more effective in reducing LDL than exercise, exercise application was more effective in increasing HDL than diet application, and combined interventions reduced total cholesterol, TG, and LDL cholesterol [51]. In this study, there was a decrease in TC, LDL, and TG levels and an increase in HDL levels in all groups except group C. The decrease in TC, LDL, and TG levels was clearly seen in group N, while the increase in HDL levels was clearly seen in group E + N. Since exercise and nutrition have an effect on blood lipids, it is thought that nutrition has a greater effect. Thus, it should be evaluated whether the risk of cardiovascular disease can be prevented through nutrition. Weight loss through exercise-induced energy expenditure was found to improve glucose tolerance and insulin action relative to similar weight loss through calorie restriction [52]. In this study, a ~38% decrease occurred in group N and a ~40% decrease occurred in group E + N. It can be said that the effect of nutrition on insulin levels is more pronounced. It was reported that body weight loss had an effect on the level of insulin resistance [53], that there was a 70% decrease in HOMA-IR levels between the groups that received calorie restriction, exercise, and both calorie restriction and exercise, and that the greatest decrease was seen in the group that had both exercise and calorie restriction [40]. In this study, it was predicted that there was a significant decrease in the pre–post-HOMA-IR levels of both groups B and E + N according to the HOMA-IR level, and in this context, the nutrition factor may be a more effective factor in fasting glucose, insulin, and HOMA-IR levels.

In studies where diet alone, exercise alone, and diet + exercise were applied, it was found that serum leptin concentrations showed significant decreases within the groups, but there were no differences between the groups [54]. Changes in leptin levels are reported to be associated with body fat mass [55]. In this study, it was thought that the greatest decrease in leptin levels occurred in group C, and in this context, weight gain or loss may have an effect on leptin levels. Additionally, it was thought that serum leptin levels can be used as an indicator to evaluate nutritional status. It was reported that ghrelin levels were not affected after acute exercise [56], but an increase in ghrelin levels was observed after chronic exercise [57]. Physical exercise is a strategy used against obesity because it reduces energy balance by increasing energy expenditure. The effects of exercise on hunger and food intake are quite controversial [58]. It was reported that the calorie deficit caused by exercise had an effect on the ghrelin level, and the increase in body fat lost with exercise, regardless of exercise intensity, reduced the ghrelin level [59]. It was reported that dietary intervention together with regular exercise can regulate the lipid profile and ghrelin and leptin levels in obese women [60].

In this study, while the greatest decrease in ghrelin levels was observed in group E + N, there was an increase in group E. It can be said that exercise and nutrition characteristics cause differences in the ghrelin level. The irisin concentration was found to be higher in active individuals than in sedentary individuals [61]. It was observed that the irisin level peaked after 3–60 min of exercise and returned to the baseline level after 6 h [62], but the irisin level did not return to the baseline level after chronic exercise ranging from 6 weeks to 1 year [63]. It was reported that the type of exercise affected the level of irisin, and the upregulation of irisin was noted after high-intensity exercise and resistance training, but no increase was reported after endurance exercise [64,65]. The effect of exercise on the irisin concentration in the blood is unclear. Exercise appears to increase circulating irisin concentrations in overweight or obese individuals. There are contrary arguments to the theory that irisin levels increase in obese conditions [66,67]. According to Pardo et al., a 1 kg increase in fat mass can lead to a two-fold increase in the irisin level [68], while different studies show that weight loss in obese individuals leads to a decrease in serum irisin levels [69,70]. Additionally, depending on the exercise type, significant differences in the irisin level are reported [71]. There are controversial results in the literature regarding irisin-related slight overweight/obesity, exercise, and dietary interventions. In this study, the irisin level decreased in group C and increased in other groups, with the highest increase occurring in group E. In this context, it is thought that the irisin level might be related to the increase in fat mass and decrease in muscle mass along with the increase in body weight seen in the control group, but the level of weight loss had no effect on the irisin level. It is suggested that exercise may play a key pathophysiological role in the regulation of the systemic energy metabolism of mitochondrial stress-induced GDF15 [72]. GDF15 levels were observed to increase slightly after a 12-week aerobic training program in obese individuals. It was reported that training-induced increases in plasma GDF15 are significantly associated with the loss of fat mass, which may indicate the potential effects of GDF15 on fat mass [73]. It was shown that while short-term overnutrition did not increase GDF15, longer periods of caloric excess increased circulating GDF15 levels [74]. In this study, the GDF15 level increased in all groups, but the highest increase was seen in group E. In this context, it is estimated that exercise may have an increasing effect on the GDF15 level. However, the relationship between GDF and nutritional status remains unclear. When exercise is continued, a decrease in oxidative stress levels was observed [75]. It was determined that long fasting periods and restricting calorie needs by 40–50% increased the level of oxidative stress [76]. The chronic ingestion of lipid-rich meals may also increase oxidative stress [77]. In this study, it was observed that there was an increase in MDA levels in group C, while there was a decrease in MDA levels in the other groups. The decrease in MDA occurred the most in group N. It is thought that eating habits affect oxidative stress. In this context, further studies are needed on the relationship between MDA levels and eating habits, regardless of obesity.

The irisin concentration initially showed a positive correlation with MDA, a marker of oxidative stress, and a negative correlation with oxygen radical absorbance capacity (ORAC), a marker of antioxidant protection, in response to an exercise test [78]. Belviranlı et al. (2016) reported that plasma irisin showed a negative correlation with MDA, suggesting that irisin levels decrease with obesity and that irisin may have antioxidant effects [79]. In our study, the correlation between irisin and MDA was found to be moderately strong in the negative direction. It was determined that there was a decrease in MDA levels due to an increase in irisin levels or that the decrease in irisin levels could be associated with an increase in MDA levels. Studies in the literature are inconsistent. In this context, it is seen in our study that irisin levels may be associated with MDA levels. More studies are needed on this subject. It has been observed that there is an increase in GDF15 levels due to the increase in body weight, but GDF15 provides an increase in GDF15 levels independently of BMI [80]. It has been determined that the decrease in BMI after applications for weight loss acutely affects GDF15 levels, and that the relationship with a chronic increase in GDF15 is contradictory [81]. In a study comparing obese and non-obese individuals, it has been reported that non-obese individuals have high GDF15 levels, and in this context, GDF15 levels are inversely proportional to BMI [82].

An increase in body weight, resulting in a rise in body fat percentage, and a decrease in body muscle percentage significantly affect quality of life. The most important key to losing excess weight is changing one’s lifestyle. This study aimed to investigate the effects of an eight-week exercise and nutrition program on blood lipids, glucose, insulin, HOMA-IR, leptin, ghrelin, irisin, MDA, and GDF15 in overweight women. It was determined that the body composition components, lipid profile indicators, insulin, glucose, insulin resistance, leptin, ghrelin, irisin, and MDA parameters examined in this study showed positive changes in the intervention groups. Additionally, exercise intervention was found to be effective on the GDF15 parameter. The greatest decrease in body weight was observed in group E + N. The decrease in body weight affected BMI, body fat percentage, degree of visceral fat, and insulin level. Group E had a greater effect on body muscle percentage, MDA, and irisin levels, while group N had a greater effect on blood lipids and ghrelin levels. Because no intervention was made to group C, an increase in body weight was observed and, accordingly, an increase was observed in BMI, BFP, VFD, W/H, and blood lipid parameters.

In our study, it has been determined that there is a decrease in BMI levels due to the increase in GDF15 levels, or that the decrease in GDF15 levels may be related to the increase in BMI levels. In our study as a whole, it is thought that although exercise has an increasing effect on GDF15 levels, its relationship with the level of body weight loss is weak, but it may be related to different parameters related to exercise. In this context, our study is similar to the literature. More studies are needed on this subject.

## 5. Strengths, Limitations, and Recommendations

It was observed that the limited change in lifestyle was effective in terms of maintaining and sustaining health, and in addition, exercise and nutrition among lifestyle changes had a holistic effect in order to achieve an effective decrease in body weight. It was observed that oxidative stress may be related to muscle mass, and muscle mass is an important factor in the evaluation of oxidative stress in exercise. In addition, it is thought that GDF15 can be developed as a new parameter for use in the diagnosis and treatment of obesity from a biochemical perspective. There is a wide variability between individuals in terms of weight loss and inadequacy in maintaining weight loss in the long term. Evidence-based dietary approaches for energy restriction that are effective in the long term are needed to provide a range of evidence-based options for individuals who want to lose weight. This study was conducted on women with a BMI of 25.0–29.9 kg/m^2^ with an 8-week follow-up, and the results obtained were limited in terms of reflecting them to the general population. It was also understood that the effects of the exercise and nutrition interventions were limited due to them being of a single type and limited in duration. In this context, it is thought that more meaningful results can be obtained in the long term by extending both the sample group and the research period by providing nutrition and exercise diversity in this study.

## 6. Conclusions

As a result, it was determined that more body weight loss was observed in the groups for which exercise and healthy nutrition were applied together, and accordingly, BMI, body fat, and muscle percentage, degree of visceral fat, the waist–hip ratio, and ghrelin levels decreased, while the HDL cholesterol levels increased, that healthy nutrition decreased blood lipids, glucose, insulin, leptin, and MDA levels, and that exercise was associated with an increase in body percentage, creatine, irisin, and GDF15 levels.

## Figures and Tables

**Table 1:** Study groups.

Control (C)	Consisted of women with a BMI of 25.0–29.9 kg/m^2^ (no nutrition program or exercise was applied for 8 weeks).
Exercise (E)	Consisted of women with a BMI of 25.0–29.9 kg/m^2^ (60–70% intensity exercise was applied 5 days a week for 8 weeks).
Nutrition (N)	Consisted of women with a BMI of 25.0–29.9 kg/m^2^ (personalized nutrition program for 8 weeks).
Exercise + nutrition (E + N)	Consisted of women with a BMI of 25.0–29.9 kg/m^2^ (60–70% intensity exercise and nutrition program was applied 5 days a week for 8 weeks).

**Table 2:** Findings regarding the anthropometric measurements of the participants.

	Groups				*p* ^1^
	C	E	N	E + N	
BW					
Before	70.97 ± 7.73	77.53 ± 8.75	76.46 ± 5.00	80.21 ± 10.03	0.131
After	72.90 ± 8.08	73.79 ± 8.60	73.19 ± 4.34	74.69 ± 10.39	0.978
Δ BW	1.94 ± 1.51 ^a^	−3.74 ± 1.02 ^b^	−3.26 ± 1.42 ^b^	−5.52 ± 1.09 ^c^	≤0.0001
*p* ^2^	0.002	≤0.0001	0.001	≤0.0001	
BMI					
Before	27.21 ± 2.66 ^b^	29.51 ± 2.09 ^ab^	28.50 ± 1.34 ^ab^	29.98 ± 1.86 ^a^	0.033
After	27.94 ± 2.68	28.11 ± 1.93	27.47 ± 1.29	28.14 ± 1.80	0.926
Δ BMI	0.73 ± 0.58 ^a^	−1.40 ± 0.38 ^b^	−1.02 ± 0.57 ^b^	−1.84 ± 0.67 ^c^	≤0.0001
*p* ^2^	0.002	≤0.0001	0.003	0.002	
BFP					
Before	35.25 ± 5.16 ^b^	37.55 ± 3.66 ^ab^	37.71 ± 2.25 ^b^	39.64 ± 0.96 ^a^	0.009
After	35.67 ± 5.01	34.49 ± 4.33	34.96 ± 2.24	35.26 ± 2.20	0.920
Δ PFP	0.43 ± 1.78 ^a^	−3.06 ± 1.86 ^b^	−2.76 ± 2.14 ^b^	−4.38 ± 1.82 ^b^	≤0.0001
*p* ^2^	0.444	≤0.0001	0.028	0.006	
BMP					
Before	61.78 ± 4.91 ^a^	59.20 ± 5.08 ^ab^	59.88 ± 2.28 ^a^	57.41 ± 1.12 ^b^	0.040
After	61.54 ± 4.66	62.33 ± 3.57	62.13 ± 2.27	61.57 ± 1.93	0.926
Δ BMP	−0.23 ± 1.53 ^b^	3.13 ± 3.44 ^a^	2.25 ± 1.82 ^ab^	4.16 ± 1.62 ^a^	0.004
*p* ^2^	0.623	0.026	0.017	0.005	
VFD					
Before	4.41 ± 1.96 ^b^	5.55 ± 1.31 ^ab^	6.79 ± 1.80 ^ab^	6.00 ± 1.06 ^a^	0.035
After	4.59 ± 1.85	4.55 ± 1.38	5.43 ± 0.53	4.60 ± 1.34	0.588
Δ VFD	0.18 ± 0.40 ^a^	−1.00 ± 0.63 ^b^	−1.36 ± 1.68 ^b^	−1.40 ± 0.65 ^b^	0.002
*p* ^2^	0.167	≤0.0001	0.076	0.009	
W/H					
Before	0.80 ± 0.05	0.81 ± 0.11	0.78 ± 0.03	0.80 ± 0.04	0.802
After	0.81 ± 0.08	0.77 ± 0.05	0.78 ± 0.03	0.78 ± 0.05	0.490
Δ W/H	0.01 ± 0.05	−0.04 ± 0.74	−0.01 ± 0.01	−0.02 ± 0.01	0.170
*p* ^2^	0.537	0.105	0.143	0.004	

BW: body weight (kg), BMI: body mass index (kg/m^2^), BFP: body fat percentage (%), BMP: body muscle percentage (%), VFD: visceral fat degree, W/H (waist circumference/hip circumference). C: control, E: exercise, N: nutrition, E + N: exercise + nutrition. ^1^ According to the normality test, the differences between the groups were examined using an ANOVA and Duncan’s test, or the differences between the groups were examined using the Kruskal–Wallis test and Mann–Whitney U test (*p* < 0.05). ^2^ According to the normality test, the differences between the groups before and after were examined with the dependent t test or Wilcoxon’s test (*p* < 0.05). The data were presented as mean and standard deviation. ^a,b,c^ the difference between the groups carrying different letters on the same line is statistically significant (*p* < 0.05). Δ: change.

**Table 3:** Findings regarding the serum biochemistry measurements of the participants.

	Groups				*p* ^1^
	C	E	N	E + N	
TC					
Before	152.09 ± 29.04 ^ab^	140.73 ± 20.00 ^b^	176.14 ± 21.11 ^a^	164.20 ± 21.98 ^ab^	0.038
After	160.90 ± 25.82 ^a^	129.00 ± 25.53 ^b^	156.86 ± 20.55 ^a^	151.80 ± 24.70 ^ab^	0.044
Δ TC	8.81 ± 5.62 ^a^	−11.72 ± 13.98 ^b^	−19.29 ± 3.09 ^b^	−12.40 ± 15.13 ^b^	≤0.0001
*p* ^2^	≤0.0001	0.021	≤0.0001	0.141	
HDL					
Before	41.55 ± 6.70	45.64 ± 6.12	43.71 ± 17.30	51.64 ± 11.67	0.201
After	41.18 ± 5.49 ^b^	48.30 ± 6.96 ^b^	50.71 ± 18.38 ^ab^	60.06 ± 9.11 ^a^	0.013
Δ HDL	−0.36 ± 1.96 ^b^	2.66 ± 3.22 ^b^	7.00 ± 3.52 ^a^	8.42 ± 6.54 ^a^	≤0.0001
*p* ^2^	0.553	0.021	0.018	0.045	
LDL					
Before	114.45 ± 23.61	87.82 ± 18.36	112.71 ± 31.46	122.20 ± 44.07	0.066
After	117.90 ± 24.36 ^a^	87.45 ± 18.84 ^b^	106.14 ± 9.50 ^ab^	117.80 ± 29.76 ^a^	0.021
Δ LDL	3.45 ± 4.48	−0.36 ± 10.11	−6.57 ± 12.19	−4.40 ± 17.39	0.235
*p* ^2^	0.029	0.907	0.204	0.602	
TG					
Before	109.09 ± 20.28	95.91 ± 25.00	111.00 ± 54.83	95.40 ± 56.30	0.326
After	111.72 ± 20.38 ^a^	89.36 ± 16.20 ^b^	97.57 ± 36.49 ^ab^	83.00 ± 24.62 ^b^	0.036
Δ TG	2.64 ± 3.93	−6.55 ± 15.98	−13.42 ± 28.09	−12.40 ± 32.49	0.322
*p* ^2^	0.059	0.204	0.253	0.686	
Glucose (fasting)					
Before	89.55 ± 12.06	83.00 ± 6.47	89.29 ± 9.91	84.60 ± 9.18	0.435
After	86.18 ± 5.60	82.82 ± 5.91	82.86 ± 5.98	78.60 ± 5.08	0.147
Δ Glucose	−3.36 ± 7.66 ^ab^	−0.18 ± 3.40 ^a^	−6.43 ± 5.32 ^b^	−6.00 ± 6.32 ^b^	0.034
*p* ^2^	0.176	0.863	0.019	0.101	
Insulin (fasting)					
Before	9.55 ± 1.65	12.54 ± 5.09	13.33 ± 3.66	12.74 ± 2.79	0.125
After	9.61 ± 1.65	10.39 ± 4.26	8.29 ± 2.01	7.61 ± 3.35	0.233
Δ Insulin	0.06 ± 0.44 ^a^	−2.15 ± 3.35 ^b^	−5.04 ± 1.75 ^b^	−5.13 ± 3.10 ^b^	≤0.0001
*p* ^2^	0.650	0.059	≤0.0001	0.043	
HOMA-IR					
Before	2.12 ± 0.55	2.59 ± 1.14	2.97 ± 0.98	2.63 ± 0.40	0.247
After	2.05 ± 0.43	2.14 ± 0.93	1.71 ± 0.49	1.46 ± 0.60	0.222
Δ HOMA-IR	−0.07 ± 0.19	−0.45 ± 0.71	−1.26 ± 0.54	−1.17 ± 0.57	≤0.0001
*p* ^2^	0.235	0.063	0.001	0.011	

TC: cholesterol (mg/dL), HDL: high-density lipoprotein cholesterol (mg/dL), LDL: low-density lipoprotein cholesterol (mg/dL), TG: triglyceride (mg/dL), glucose (mg/dL), insulin (μIU/mL), HOMA-IR: homeostatic model assessment insulin resistance. C: control, E: exercise, N: nutrition, E + N: exercise + nutrition. ^1^ According to the normality test, the differences between the groups were examined using an ANOVA and the Duncan test, or the differences between the groups were examined using the Kruskal–Wallis test and Mann–Whitney U test (*p* < 0.05). ^2^ According to the normality test, the differences between the groups before and after were examined with the dependent t test or Wilcoxon’s test (*p* < 0.05). The data were presented as the mean and standard deviation. ^a,b,c^ The difference between the groups carrying different letters on the same line is statistically significant (*p* < 0.05). Δ: change.

**Table 4:** Findings regarding the serum parameters of the participants.

	Groups				*p* ^1^
	C	E	N	E + N	
Leptin					
Before	50.12 ± 3.82 ^a^	42.40 ± 2.68 ^ab^	50.52 ± 3.78 ^a^	37.78 ± 6.95 ^b^	0.045
After	35.01 ± 1.18 ^b^	38.94 ± 1.47 ^ab^	41.10 ± 2.64 ^a^	34.20 ± 1.10 ^b^	0.027
Δ Leptin	−15.11 ± 8.12	−3.47 ± 8.80	−9.42 ± 11.20	−3.58 ± 6.86	0.162
*p* ^2^	0.014	0.428	0.133	0.308	
Ghrelin					
Before	713.43 ± 83.41	654.99 ± 48.70	649.08 ± 62.07	690.99 ± 80.00	0.440
After	648.39 ± 144.62 ^ab^	685.80 ± 54.38 ^a^	520.00 ± 36.29 ^c^	548.98 ± 64.79 ^bc^	0.022
Δ Ghrelin	−65.04 ± 221.99	30.80 ± 80.97	−129.08 ± 42.72	−143.01 ± 136.83	0.219
*p* ^2^	0.548	0.443	0.003	0.080	
İrisin					
Before	34.45 ± 2.93 ^b^	29.04 ± 4.24 ^c^	32.39 ± 2.06 ^bc^	39.24 ± 4.13 ^a^	0.002
After	31.83 ± 1.84 ^b^	46.13 ± 4.94 ^a^	45.02 ± 5.47 ^a^	41.75 ± 4.27 ^a^	≤0.0001
Δ İrisin	−2.62 ± 4.46 ^b^	17.09 ± 5.09 ^a^	12.63 ± 6.72 ^a^	2.51 ± 4.75 ^b^	≤0.0001
*p* ^2^	0.259	0.002	0.014	0.303	
GDF15					
Before	368.80 ± 42.10 ^a^	266.50 ± 23.31 ^b^	329.53 ± 12.81 ^a^	280.43 ± 35.36 ^b^	≤0.0001
After	375.51 ± 39.76 ^a^	295.36 ± 25.53 ^b^	332.24 ± 51.61 ^ab^	309.24 ± 24.80 ^b^	0.018
Δ GDF15	6.71 ± 69.95	28.85 ± 28.74	2.71 ± 60.58	28.81 ± 40.00	0.801
*p* ^2^	0.841	0.088	0.925	0.259	
MDA					
Before	0.86 ± 0.15	1.13 ± 0.26	1.02 ± 0.28	0.84 ± 0.39	0.365
After	0.91 ± 0.17	0.68 ± 0.37	0.51 ± 0.32	0.59 ± 0.26	0.184
Δ MDA	0.05 ± 0.30	−0.44 ± 0.26	−0.51 ± 0.44	−0.25 ± 0.36	0.089
*p* ^2^	0.736	0.018	0.062	0.190	

Leptin (ng/mL), ghrelin (pg/mL), irisin (ng/mL), GDF15: Growth Differentiation Factor 15 (ng/L), MDA: malondialdehyde (nmol/mL). C: control, E: exercise, N: nutrition, E + N: exercise + nutrition. ^1^ According to the normality test, the differences between the groups were examined using an ANOVA and Duncan’s test, or the differences between the groups were examined using the Kruskal–Wallis test and Mann–Whitney U test (*p* < 0.05). ^2^ According to the normality test, the differences between the groups before and after were examined with the dependent t test or Wilcoxon’s test (*p* < 0.05). The data were presented as the mean and standard deviation. ^a,b,c^ The difference between the groups carrying different letters on the same line is statistically significant (*p* < 0.05). Δ: change.

## Data Availability

The data presented in this study are available upon request from the corresponding authors.

## References

[1] Austin J., Marks D. (2009). Hormonal regulators of appetite. Int. J. Pediatr. Endocrinol..

[2] Heianza Y., Qi L. (2017). Gene-Diet interaction and precision nutrition in obesity. Int. J. Mol. Sci..

[3] Tchang B.G., Saunders K.H., Igel L.I. (2021). Best practices in the management of overweight and obesity. Med. Clin. N. Am..

[4] Amin M.N., Siddiqui S.A., Uddin G., Ibrahim, Uddin S.M.N., Adnan T., Rahaman Z., Kar A., Islam M.S. (2020). Increased Oxidative Stress, Altered Trace Elements, and Macro-Minerals Are Associated with Female Obesity. Biol. Trace Elem. Res..

[5] Said M., Lamya N., Olfa N., Hamda M. (2017). Effects of high-impact aerobics vs. low-impact aerobics and strength training in overweight and obese women. J. Sports Med. Phys. Fit..

[6] Ismail I., Keating S.E., Baker M.K., Johnson N.A. (2012). A systematic review and meta-analysis of the effect of aerobic vs. resistance exercise training on visceral fat. Obes. Rev..

[7] Fonseca-Junior S.J., Sá C.G., Rodrigues P.A., Oliveira A.J., Fernandes-Filho J. (2013). Physical exercise and morbid obesity: A systematic review. Arq. Bras. Cir. Dig..

[8] Racil G., Russo L., Migliaccio G.M., Signorelli P., Larion A., Padulo J., Jlid M.C. (2023). High-Intensity Interval Training in Female Adolescents with Moderate or Severe Obesity. Children.

[9] Racil G., Chelly M.-S., Coquart J., Padulo J., Teodor D.F., Russo L. (2023). Long- and Short-Term High-Intensity Interval Training on Lipid Profile and Cardiovascular Disorders in Obese Male Adolescents. Children.

[10] World Health Organization (2013). Global Physical Activity Questionnaire (GPAQ) Analysis Guide 2011.

[11] Oja P., Titze S. (2011). Physical activity recommendations for public health: Development and policy context. EPMA J..

[12] Yusuf S., Hawken S., Ôunpuu S., Dans T., Avezum A., Lanas F., McQueen M., Budaj A., Pais P., Varigos J. (2004). Effect of potentially modifiable risk factors associated with myocardial infarction in 52 countries (the INTERHEART study): Case-control study. Lancet.

[13] Csige I., Ujvárosy D., Szabó Z., Lőrincz I., Paragh G., Harangi M., Somodi S. (2018). The Impact of Obesity on the Cardiovascular System. J. Diabetes Res..

[14] Mika A., Sledzinski T. (2017). Alterations of specific lipid groups in serum of obese humans: A review. Obes. Rev..

[15] Ahmed B., Sultana R., Greene M.W. (2021). Adipose tissue and insulin resistance in obese. Biomed. Pharmacother..

[16] Topsakal S., Yerlikaya E., Akin F., Kaptanoglu B., Erürker T. (2012). Relation with HOMA-IR and thyroid hormones in obese Turkish Women with Metabolic Syndrome. Eat. Weight. Disord..

[17] Normandin E., Chmelo E., Lyles M.F., Marsh A.P., Nicklas B.J. (2017). Effect of Resistance Training and Caloric Restriction on the Metabolic Syndrome. Med. Sci. Sports Exerc..

[18] Cummings D.E., Purnell J.Q., Frayo R.S., Schmidova K., Wisse B.E., Weigle D.S. (2001). A preprandial rise in plasma ghrelin levels suggests a role in meal initiation in humans. Diabetes.

[19] Mai S., Grugni G., Mele C., Vietti R., Vigna L., Sartorio A., Aimaretti G., Scacchi M., Marzullo P. (2020). Irisin levels in genetic and essential obesity: Clues for a potential dual role. Sci. Rep..

[20] Mullican S.E., Rangwala S.M. (2018). Uniting GDF15 and GFRAL: Therapeutic Opportunities in Obesity and Beyond. Trends Endocrinol. Metab. TEM.

[21] Tsai V.W., Husaini Y., Sainsbury A., Brown D.A., Breit S.N. (2018). The MIC-1/GDF15-GFRAL pathway in energy homeostasis: Implications for obesity, cachexia, and other associated diseases. Cell Metab..

[22] Kim K.H., Kim S.H., Han D.H., Jo Y.S., Lee Y.H., Lee M.S. (2018). Growth differentiation factor 15 ameliorates nonalcoholic steatohepatitis and related metabolic disorders in mice. Sci. Rep..

[23] Gough D.R., Cotter T.G. (2011). Hydrogen peroxide: A Jekyll and Hyde signalling molecule. Cell Death Dis..

[24] Neha K., Haider M.R., Pathak A., Yar M.S. (2019). Medicinal prospects of antioxidants: A review. Eur. J. Med. Chem..

[25] Teng N.I., Shahar S., Rajab N.F., Manaf Z.A., Johari M.H., Ngah W.Z. (2013). Improvement of metabolic parameters in healthy older adult men following a fasting calorie restriction intervention. Aging Male..

[26] Faul F., Erdfelder E., Lang A.G., Buchner A. (2007). G* Power 3: A flexible statistical power analysis program for the social, behavioral, and biomedical sciences. Behav. Res. Methods.

[27] Wirth A., Wabitsch M., Hauner H. (2014). The prevention and treatment of obesity. Dtsch. Arztebl. Int..

[28] Karvonen M.J., Kentala E., Mustala O. (1957). The effects of training on heart rate; A longitudinal study. Ann. Med. Exp. Biol. Fenn..

[29] Karvonen M.J., Vuorimaa T. (1988). Heart rate and exercise intensity during sports activities: Practical application. Sports Med..

[30] Baysal A. (1998). Sağlikli Beslenme: Uzmanların önerisi tüketicinin algılaması. Beslenme Diyet Derg. J. Nutr. Diet..

[31] Baysal A., Aksoy M., Besler H.T., Bozkurt N., Keçeçioğlu S., Mercanlıgil S.M., Kutluay-Merdol T., Pekcan G., Yıldız E. (2014). Diyet El Kitabı (8. Baskı).

[32] Yumuk V., Tsigos C., Fried M., Schindler K., Busetto L., Micic D., Toplak H. (2015). Obesity Management Task Force of the European Association for the Study of Obesity. European Guidelines for Obesity Management in Adults. Obes. Facts.

[33] Harris J.A., Benedict F.G. (1918). A biometric study of human basal metabolism. Proc. Natl. Acad. Sci. USA.

[34] Pekcan G. (2008). Beslenme Durumunun Saptanması. Diyet Kitabı. Ankara.

[35] (2022). Türkiye Beslenme Rehberi (TÜBER). https://hsgm.saglik.gov.tr/depo/birimler/saglikli-beslenme-ve-hareketli-hayat-db/Dokumanlar/Rehberler/Turkiye_Beslenme_Rehber_TUBER_2022_min.pdf.

[36] Rakıcıoğlu N., Tek N., Ayaz A., Pekcan G. (2014). Yemek ve Besin Fotoğraf Kataloğu.

[37] Gür M., Çınar V., Akbulut T., Bozbay K., Yücedal P., Aslan M., Avcu G., Padulo J., Russo L., Rog J. (2024). Determining the Levels of Cortisol, Testosterone, Lactic Acid and Anaerobic Performance in Athletes Using Various Forms of Coffee. Nutrients.

[38] Bengü A.Ş. (2021). HPLC Tekniği ve Kullanım Alanları. BÜSAD.

[39] IBM SPSS (2012). IBM Corp. Released 2012. IBM SPSS Statistics for Windows, Version 22.0.

[40] Weiss E.P., Albert S.G., Reeds D.N., Kress K.S., McDaniel J.L., Klein S., Villareal D.T. (2016). Effects of matched weight loss from calorie restriction, exercise, or both on cardiovascular disease risk factors: A randomized intervention trial. Am. J. Clin. Nutr..

[41] Garvey W.T., Mechanick J.I., Brett E.M., Garber A.J., Hurley D.L., Jastreboff A.M., Nadolsky K., Pessah-Pollack R., Plodkowski R. (2016). Reviewers of the AACE/ACE Obesity Clinical Practice Guidelines. Off. J. Am. Coll. Endocrinol. Am. Assoc. Clin. Endocrinol..

[42] Seo Y.-G., Lim H., Kim Y., Ju Y.-S., Lee H.-J., Jang H.B., Park S.I., Park K.H. (2019). The Effect of a Multidisciplinary Lifestyle Intervention on Obesity Status, Body Composition, Physical Fitness, and Cardiometabolic Risk Markers in Children and Adolescents with Obesity. Nutrients.

[43] Correa-Rodríguez M., Rueda-Medina B., González-Jiménez E., Schmidt-RioValle J. (2017). Associations between body composition, nutrition, and physical activity in young adults. Am. J. Hum. Biol..

[44] Swainson M.G., Batterham A.M., Tsakirides C., Rutherford Z.H., Hind K. (2017). Prediction of whole-body fat percentage and visceral adipose tissue mass from five anthropometric variables. PLoS ONE.

[45] Oliveros E., Somers V.K., Sochor O., Goel K., Lopez-Jimenez F. (2014). The concept of normal weight obesity. Prog. Cardiovasc. Dis..

[46] Bergman R.N., Stefanovski D., Buchanan T.A., Sumner A.E., Reynolds J.C., Sebring N.G., Xiang A.H., Watanabe R.M. (2011). A better index of body adiposity. Obesity.

[47] Arner P., Andersson D.P., Bäckdahl J., Dahlman I., Rydén M. (2018). Weight gain and impaired glucose metabolism in women are predicted by inefficient subcutaneous fat cell lipolysis. Cell Metab..

[48] Carbone J.W., Pasiakos S.M. (2019). Dietary Protein and Muscle Mass: Translating Science to Application and Health Benefit. Nutrients.

[49] Mahan L.K., Raymond J.L. (2016). Krause’s Food & The Nutrition Care Process.

[50] Bozbay K., Çinar V., Akbulut T., Aydemir I., Yasul Y., Aytac K.Y., Ozkaya A., Russo L., Fusco A., Migliaccio G.M. (2024). Effects of Exercise and Pomegranate–Black Carrot Juice Interventions on Mineral Metabolism and Fatty Acids. Appl. Sci..

[51] Khalafi M., Sakhaei M.H., Kazeminasab F., Rosenkranz S.K., Symonds M.E. (2023). Exercise training, dietary intervention, or combined interventions and their effects on lipid profiles in adults with overweight and obesity: A systematic review and meta-analysis of randomized clinical trials. Nutr. Metab. Cardiovasc. Dis..

[52] Weiss E.P., Racette S.B., Villareal D.T., Fontana L., Steger-May K., Schechtman K.B., Klein S., O Holloszy J., Washington University School of Medicine CALERIE Group (2006). Improvements in glucose tolerance and insulin action induced by increasing energy expenditure or decreasing energy intake: A randomized controlled trial. Am. J. Clin. Nutr..

[53] Han L., Zhang T., You D., Chen W., Bray G., Sacks F., Qi L. (2022). Temporal and mediation relations of weight loss, and changes in insulin resistance and blood pressure in response to 2-year weight-loss diet interventions: The POUNDS Lost trial. Eur. J. Nutr..

[54] Volpe S.L., Kobusingye H., Bailur S., Stanek E. (2008). Effect of diet and exercise on body composition, energy intake and leptin levels in overweight women and men. J. Am. Coll. Nutr..

[55] Thong F.S., Hudson R., Ross R., Janssen I., Graham T.E. (2000). Plasma leptin in moderately obese men: Independent effects of weight loss and aerobic exercise. Am. J. Physiol. Endocrinol. Metab..

[56] Broom D.R., Stensel D.J., Bishop N.C., Burns S.F., Miyashita M. (2007). Exercise-induced suppression of acylated ghrelin in humans. J. Appl. Physiol..

[57] Yu A.P., Ugwu F.N., Tam B.T., Lee P.H., Lai C.W., Wong C.S.C., Lam W.W., Sheridan S., Siu P.M., Yu A.P. (2018). One Year of Yoga Training Alters Ghrelin Axis in Centrally Obese Adults with Metabolic Syndrome. Front. Physiol..

[58] Vatansever-Ozen S., Tiryaki-Sonmez G., Bugdayci G., Ozen G. (2011). The effects of exercise on food intake and hunger: Relationship with acylated ghrelin and leptin. J. Sports Sci. Med..

[59] Flack K.D., Hays H.M., Moreland J., Long D.E. (2020). Exercise for Weight Loss: Further Evaluating Energy Compensati, on with Exercise. Med. Sci. Sports Exerc..

[60] Bengin E., Kırtepe A., Çınar V., Akbulut T., Russo L., Aydemir I., Yücedal P., Aydın S., Migliaccio G.M. (2024). Leptin, Ghrelin, Irisin, Asprosin and Subfatin Changes in Obese Women: Effect of Exercise and Different Nutrition Types. Medicina.

[61] Moreno M., Moreno-Navarrete J.M., Serrano M., Ortega F., Delgado E., Sanchez-Ragnarsson C., Valdés S., Botas P., Ricart W., Fernández-Real J.M. (2015). Circulating irisin levels are positively associated with metabolic risk factors in sedentary subjects. PLoS ONE.

[62] Nygaard H., Slettaløkken G., Vegge G., Hollan I., Whist J.E., Strand T., Rønnestad B.R., Ellefsen S. (2015). Irisin in blood increases transiently after single sessions of intense endurance exercise and heavy strength training. PLoS ONE.

[63] Moraes C., Leal V.O., Marinho S.M., Barroso S.G., Rocha G.S., Boaventura G.T., Mafra D. (2013). Resistance exercise training does not affect plasma irisin levels of hemodialysis patients. Horm. Metab. Res..

[64] Tsuchiya Y., Ando D., Goto K., Kiuchi M., Yamakita M., Koyama K. (2014). High-intensity exercise causes greater irisin response compared with low-intensity exercise under similar energy consumption. Tohoku, J. Exp. Med..

[65] Tsuchiya Y., Ando D., Takamatsu K., Goto K. (2015). Resistance exercise induces a greater irisin response than endurance exercise. Metabolism.

[66] Sahin-Efe A., Upadhyay J., Ko B.J., Dincer F., Park K.H., Migdal A., Vokonas P., Mantzoros C. (2018). Irisin and leptin concentrations in relation to obesity, and developing type 2 diabetes: A cross sectional and a prospective case-control study nested in the Normative Aging Study. Metabolism.

[67] De Meneck F., de Souza L.V., Oliveira V., do Franco M.C. (2018). High irisin levels in overweight/obese children and its positive correlation with metabolic profile, blood pressure, and endothelial progenitor cells. Nutr. Metab. Cardiovasc. Dis..

[68] Pardo M., Crujeiras A.B., Amil M., Aguera Z., Jiménez-Murcia S., Baños R., Botella C., de la Torre R., Estivill X., Fagundo A.B. (2014). Association of irisin with fat mass, resting energy expenditure, and daily activity in conditions of extreme body mass index. Int. J. Endocrinol..

[69] Crujeiras A.B., Zulet M.A., Lopez-Legarrea P., de la Iglesia R., Pardo M., Carreira M.C., Martinez J.A., Casanueva F.F. (2014). Association between circulating irisin levels and the promotion of insulin resistance during the weight maintenance period after a dietary weight-lowering program in obese patients. Metabolism.

[70] Huerta A.E., Prieto-Hontoria P.L., Fernandez-Galilea M., Sainz N., Cuervo M., Martinez J.A., Moreno-Aliaga M.J. (2015). Circulating irisin and glucose metabolism in overweight/obese women: Effects of α-lipoic acid and eicosapentaenoic acid. J. Physiol. Biochem..

[71] Akbulut T., Cinar V., Aydin S., Yardim M. (2022). The role of different exercises in irisin, heat shock protein 70 and some biochemical parameters. J. Med. Biochem..

[72] Ost M., Igual Gil C., Coleman V., Keipert S., Efstathiou S., Vidic V., Weyers M., Klaus S. (2020). Muscle-derived GDF15 drives diurnal anorexia and systemic metabolic remodeling during mitochondrial stress. EMBO Rep..

[73] Zhang H., Fealy C.E., Kirwan J.P. (2019). Exercise training promotes a GDF15-associated reduction in fat mass in older adults with obesity. Am. J. Physiol. Endocrinol. Metab..

[74] Patel S., Alvarez-Guaita A., Melvin A., Rimmington D., Dattilo A., Miedzybrodzka E.L., Cimino I., Maurin A.C., Roberts G.P., Meek C.L. (2019). GDF15 Provides an Endocrine Signal of Nutritional Stress in Mice and Humans. Cell Metab..

[75] Deminice R., Sicchieri T., Mialich M.S., Milani F., Ovidio P.P., Jordao A.A. (2011). Oxidative stress biomarker responses to an acute session of hypertrophy-resistance traditional interval training and circuit training. J. Strength Cond. Res..

[76] Stankovic M., Mladenovic D., Ninkovic M., Vucevic D., Tomasevic T., Radosavljevic T. (2013). Effects of caloric restriction on oxidative stress parameters. Gen. Physiol. Biophys..

[77] Bloomer R.J., Kabir M.M., Marshall K.E., Canale R.E., Farney T.M. (2010). Postprandial oxidative stress in response to dextrose and lipid meals of differing size. Lipids Health Dis..

[78] Joro R., Korkmaz A., Lakka T.A., Uusitalo A.L.T., Atalay M. (2020). Plasma irisin and its associations with oxidative stress in athletes suffering from overtraining syndrome. Physiol. Int..

[79] Belviranli M., Okudan N., Çelik F. (2016). Association of Circulating Irisin with Insulin Resistance and Oxidative Stress in Obese Women. Horm. Metab. Res..

[80] Hong J.H., Chung H.K., Park H.Y., Joung K.-H., Lee J.H., Jung J.G., Kim K.S., Kim H.J., Ku B.J., Shong M. (2014). GDF15 Is a Novel Biomarker for Impaired Fasting Glucose. Diabetes Metab. J..

[81] Karhunen V., Larsson S.C., Gill D. (2021). Genetically proxied growth-differentiation factor 15 levels and body mass index. Br. J. Clin. Pharmacol..

[82] Hale C., Veniant M.M. (2020). Growth differentiation factor 15 as a potential therapeutic for treating obesity. Mol. Metab..

